# Identification of cytotoxic T cells and their T cell receptor sequences targeting COVID-19 using MHC class I-binding peptides

**DOI:** 10.1038/s10038-022-01013-4

**Published:** 2022-02-02

**Authors:** Tetsuro Hikichi, Michiko Sakamoto, Makiko Harada, Maki Saito, Yuka Yamane, Kimihisa Tokumura, Yusuke Nakamura

**Affiliations:** 1 OncoTherapy Science, Inc, Kawasaki, Kanagawa Japan; 2 Cancer Precision Medicine Center, Japanese Foundation for Cancer Research, Tokyo, Japan

**Keywords:** Cellular immunity, Sequencing

## Abstract

Since severe acute respiratory syndrome coronavirus 2 (SARS-CoV-2, COVID-19) was first reported in China in December 2019, various variants have been identified in different areas of the world such as United Kingdom (alpha), South Africa (beta and omicron), Brazil (gamma), and India (delta). Some of SARS-CoV-2 variants, each of which is characterized by a unique mutation(s) in spike protein, are concerned due to their high infectivity and the capability to escape from neutralizing antibodies elicited by vaccinations. To identify peptide epitopes that are derived from SARS-CoV-2 viral proteins and possibly induce CD8^+^ T cell immunity, we investigated SARS-CoV-2-derived peptides that are likely to bind to major histocompatibility complex (MHC) class I molecules. We identified a total of 15 peptides that bind to human leukocyte antigen (HLA)-A*24:02, HLA-A*02:01, or HLA-A*02:06, and possibly induce cytotoxic T lymphocytes (CTLs); thirteen of them corresponded to ORF1ab polyprotein, one peptide to spike protein and the remaining one to membrane glycoprotein. CD8^+^ T cells that recognize these peptides were detected in peripheral blood samples in three individuals recovered from COVID-19 as well as non-infected individuals. Since most of these peptides are commonly conserved among other coronaviruses including SARS-CoV and/or MERS-CoV, these might be useful to maintain T cell responses to coronaviruses that are pandemic at present and will become the future threat. We could define pairs of TRA and TRB sequences of nine CTL clones that recognize SARS-CoV-2-derived peptides. We might use these SARS-CoV-2-derived peptide-reactive TCR sequences for investigating the history of SARS-CoV-2 infection.

## Introduction

Severe acute respiratory syndrome coronavirus 2 (SARS-CoV-2) infection has become world-wide pandemic. Until December 21, 2021, ~273 million people have been confirmed the infection to COVID-19 and more than 5 million died of this disease.

In addition to intensive screening of infected individuals with PCR and/or antigen tests and subsequent isolation of corona-positive individuals, worldwide vaccination is fundamentally essential to overcome the present pandemic situation. Randomized trials demonstrated that Pfizer/BioNTech BNT162b2 and Moderna mRNA-1273 vaccines encoding the SARS-CoV-2 spike protein had ~95% protective ability against COVID-19 [1, 2]. Vaccine-elicited neutralizing antibodies inhibit the spike protein on SARS-CoV-2, which was originally reported in December 2019 in China, to attach to the host cells. While these vaccines against COVID-19 have been used widely, SARS-CoV-2 variants have continuously emerged in different areas of the world. Virus variants, alpha, beta, gamma, delta, and omicron, which were defined by a unique amino acid substitution(s) within the spike protein, were identified in the United Kingdom [3], South Africa [4, 5], Brazil [6], and India [7], respectively. Neutralizing activity against these SARS-CoV-2 variants tends to decrease as compared to that against the original reference strain in individuals who received vaccination [8–12]; therefore, a novel vaccine(s) that is not affected by mutations in the virus genome is required for the fight against SARS-CoV-2.

Although most of the studies have focused on the neutralizing antibodies against the spike protein, we here describe induction of CD8^+^ T cells that are expected to play crucial roles in virus clearance through killing of virus-infected cells. In fact, in SARS-CoV-2 infections, the presence of CD8^+^ T cells responding to SARS-CoV-2-derived peptides was shown to be associated with better clinical outcomes [13–15]. CD8^+^ T cells in COVID-19-recovered individuals could recognize not only spike protein-derived peptides but also other viral proteins including nucleocapsid phosphoprotein, membrane glycoprotein, and ORFs-derived peptides [16–18]. In previous studies, severe acute respiratory syndrome coronavirus (SARS-CoV)-specific memory T cells were detected in blood samples of SARS-recovered individuals after 6–11 years of the initial infection [19, 20]. T cell responses against the nucleocapsid phosphoprotein of SARS-CoV were maintained in individuals with the history of SARS-CoV infection 17 years after the outbreak of SARS in 2003 and these memory T cells showed cross-reactivity to SARS-CoV-2 [17]. These data indicate that memory T cells may also critically contribute to long-term protection from COVID-19.

In a present study, we demonstrate major histocompatibility complex (MHC) class I-binding epitope peptides derived from SARS-CoV-2 viral proteins to enhance antiviral CD8^+^ T cells immunity. To characterize cytotoxic T lymphocytes (CTLs) that recognize SARS-CoV-2-derived peptides and play a central role in T cell immunity, we further conduct T cell receptor (TCR) analysis for peptide-specific CTLs. The results should provide useful information for T cell responses against SARS-CoV-2 infection.

## Materials and methods

### Samples

The study protocol was approved by the Institutional Review Board of OncoTherapy Science and the written informed consent was obtained from each of peripheral blood mononuclear cells (PBMCs) donors.

### PBMCs

For in vitro CTL induction, human leukocyte antigen (HLA)-A*24:02-positive PBMCs were isolated from blood of healthy volunteers by density gradient centrifugation using Ficoll-Paque PLUS (Cytiva) according to an industrial instruction manual. HLA-A*02:01- or HLA-A*02:06-positive PBMCs were purchased from Cellular Technology Limited (Cleveland, OH). For tetramer staining, HLA-A*24:02-positive PBMCs derived from COVID-19-recovered individuals with a prior positive SARS-CoV-2 PCR test were purchased from Precision For Medicine (Bethesda, MD). Healthy individuals-derived PBMCs that were collected before the SARS-CoV-2 pandemic (March 2018–May 2019) were purchased from Cellular Technology Limited.

### Cell lines

TISI cells (HLA-A*24:02/-, lymphoblastoid cell) were purchased from the IHWG Cell and Gene Bank (Seattle, WA). T2 (HLA-A*02:01/-, lymphoblast), Jiyoye (HLA-A32, Burkitt’s lymphoma), and EB-3 (HLA-A3/Aw32, Burkitt’s lymphoma) cells were purchased from American Type Culture Collection (Manassas, VA). HEV0011 (HLA-A*02:06/-, B lymphocytes) cells were purchased from RIKEN Bioresource Research Center (Tsukuba, Japan). All cells were cultured in RPMI1640 media (GIBCO) supplemented with 10% fetal bovine serum (GIBCO) and 1% antibiotic solution (Wako).

### Peptides

SARS-CoV-2-derived 9-mer and 10-mer peptides were synthesized by Cosmo Bio Co., Ltd. (Otaru, Japan). The purity (>90%) and the sequences of peptides were confirmed by analytical HPLC and a mass spectrometry analysis. Peptides were dissolved in dimethyl sulfoxide at 20 mg/mL and stored at −80 °C until the use for in vitro CTL induction, an IFN-γ enzyme-linked immunospot (ELISPOT) assay and an IFN-γ enzyme-linked immunosorbent assay (ELISA).

### in vitro CTL induction

Monocyte-derived dendritic cells (DCs) were generated from PBMCs. Monocytes were isolated from PBMCs by adherence to a plastic tissue culture dish (Becton Dickinson) and cultured in the presence of 1000 IU/mL of granulocyte-macrophage colony-stimulating factor (R&D System) and 1000 IU/mL of interleukin (IL)−4 (R&D System) in AIM-V medium (Invitrogen) supplemented with 2% human AB serum (SIGMA) for 7 days. On day 5, OK-432 (Chugai Pharmaceutical) was added in the culture medium to induce the maturation of DCs (final concentration: 0.1 KE/mL). Autologous CD8^+^ T cells (3.0 × 10^5^ cells) purified from PBMCs with CD8-Positive Isolation Kit (Dynal) were cultured with DCs (1.5 × 10^4^ cells) with 20 μg/mL of each peptide in AIM-V medium containing 2% human AB serum, 10 ng/mL of IL-7 (R&D System), and 30 ng/mL of IL-21 (Cell Genix). After 3 days of the culture, CD8^+^ T cells were restimulated with DCs and 20 μg/mL of each peptide. DCs were prepared by the same procedure described above. At day 7 of the culture, CD8^+^ T cells were further stimulated with 48 IU/mL of IL-2 (Novartis), 5 ng/mL of IL-7 (Novoprotein), and 5 ng/mL of IL-15 (Novoprotein) in AIM-V medium supplemented with 2% human AB serum. At day 11 of the culture, CD8^+^ T cell responses were examined by an IFN-γ ELISPOT assay.

### IFN-γ ELISPOT assay

The human IFN-γ ELISPOT kit and AEC substrate set (BD Biosciences) were used to analyze CD8^+^ T cell responses to SARS-CoV-2-derived peptides. The ELISPOT assay was performed according to the industrial instruction manual. Briefly, TISI, T2, or HEV0011 cells were used as stimulator cells. These cells were cultured with or without 20 μg/mL of peptide overnight at 37 °C. To analyze CD8^+^ T cell responses, 500 μL of supernatant was removed from each well of culture plates for in vitro CTL induction. Subsequently 100 μL of cell suspensions and stimulator cells (2 × 10^4^ cells) with or without each peptide were co-cultured in 96-well plates coated with anti-IFN-γ antibody. After 16–18 h incubation, IFN-γ spots were detected and counted using ImmunoSpot S6 analyzer (Cellular Technology Limited).

### Limiting dilution

CD8^+^ T cells were diluted to 0.5 cell or 10 cells per well in 96-well round-bottom plates (Corning) and cultured with feeder cells in AIM-V medium containing 5% human AB serum, 30 ng/mL of anti-CD3 monoclonal antibody (clone UCHT1; BD Biosciences), and 150 IU/mL of IL-2. Jiyoye and EB-3 cells (1 × 10^4^ cells each) were used as feeder cells after treating with 30 μg/mL of Mitomycin C (MEDAC) at 37 °C for 30 min. After 10 days of the culture, IL-2 was added to each well (final concentration: 150 IU/mL). After additional 4 days of the culture, IFN-γ ELISPOT was performed for screening CD8^+^ T cell responses against each peptide.

### CTL expansion culture

CTLs that showed positive reactivity in the IFN-γ ELISPOT assay after limiting dilution were expanded. They were cultured with feeder cells in AIM-V medium containing 5% human AB serum and 40 ng/mL of anti-CD3 monoclonal antibody. Jiyoye and EB-3 cells (5 × 10^6^ cells each) were used as feeder cells after treating with 30 μg/mL of Mitomycin C at 37 °C for 30 min. The half volume of culture medium was exchanged with fresh AIM-V containing 5% human AB serum and 72 IU/mL of IL-2 every 3 or 4 days. After 14 days of the culture, IFN-γ secretion from CTLs was measured by ELISA.

### IFN-γ ELISA

ELISA were performed using IFN-γ ELISA kit (BD Biosciences) according to the industrial instruction manual. Briefly, CTLs (Responders) and TISI, T2, or HEV0011 cells (Stimulators) with or without each peptide were co-cultured in 200 μL of AIM-V medium supplemented with 5% human AB serum at several ratio of Responders to Stimulators (R/S ratio). Responders at 5 × 10^4^, 2.5 × 10^4^, 1.25 × 10^4^, 0.625 × 10^4^, or 0.3125 × 10^4^ cells/well concentration were plated in 96-well round-bottom plates together with 1 × 10^4^ Stimulators. After 16–18 h of the incubation, supernatants were collected for measurement of IFN-γ. IFN-γ production was calculated as pg/mL using a standard curve.

### TCR sequencing

Total RNAs were extracted from peptide-specific CTL clones using RNeasy mini kit (QIAGEN). cDNAs were synthesized using SMARTScribe Reverse Transcriptase (Clontech). SARS-CoV-2-derived peptides-reactive TCR sequences were identified by Sanger sequencing. TCRα cDNA was amplified by PCR using a common forward primer for adaptor (5'-GTCTCGTGGGCTCGGAGATGTGTATAAGAGACAGTATCAACGCAGAGTGGCCAT-3') and a reverse primer specific for the constant region (5'-TCGTCGGCAGCGTCAGATGTGTATAAGAGACAGDBDHHCAGGGTCAGGGTTCTGGATA-3'). Sanger sequencing was performed using M13 forward primer (5'-TGTAAAACGACGGCCAGTG-3') and M13 reverse primer (5'-CAGGAAACAGCTATGACCAT-3') after TA cloning. TCRβ cDNA was amplified using a common forward primer for adaptor and a reverse primer specific for the constant region (5'-TCGTCGGCAGCGTCAGATGTGTATAAGAGACAGDVHDVTCTGATGGCTCAAACACAGC-3') [21].

### in vitro stimulation

PBMCs (5 × 10^5^ cells/mL) obtained from COVID-19-recovered individuals or healthy individuals without the history of SARS-CoV-2 infection were stimulated with 10 μg/mL of each peptide in mixture of 50% AIM-V and 50% RPMI1640 medium supplemented with 10% fetal bovine serum. After 4 days of the stimulation, PBMCs were restimulated with 10 μg/mL of each peptide. PBMCs were cultured for 12 days adding 120 IU/mL of IL-2 on days 5, 7, and 10.

### Preparation of tetramers

SARS-CoV-2-derived peptides-loaded MHC class I (pMHCI) tetramers were generated by QuickSwitch^TM^ Quant HLA-A*24:02 Tetramer Kit-PE (MBL International Corporation) according to an industrial instruction manual [22]. Briefly, 50 μL of tetramer folded with an irrelevant exchangeable peptide at 50 μg/mL was mixed with 1 μL of peptide at 1 mg/mL and 1 μL of Peptide Exchange Factor for 4 h at room temperature. Rate of peptide exchange was quantitated by flow cytometry. When irrelevant exchangeable peptides were exchanged for SARS-CoV-2-derived peptides at a rate of more than 75%, tetramers were used for staining PBMCs.

### Tetramer staining

After in vitro stimulation, PBMCs were incubated with phycoerythrin (PE)-conjugated tetramer on ice for 30 min. Following wash in Dulbecco’s phosphate-buffered saline (DPBS, GIBCO) with 0.5% bovine serum albumin (BSA, Iwai Chemicals), the cells were stained with fluorescein isothiocyanate-conjugated anti-human CD8 antibody (clone RPA-T8, BD Biosciences), allophycocyanin-conjugated anti-human CD3 antibody (clone UCHT1, BD Biosciences), and phycoerythrin-Cy7 (PE-Cy7)-conjugated anti-human CD4 antibody (clone RPA-T4, BD Biosciences) on ice for 20 min. The cells were washed in DPBS with 0.5% BSA and then stained with DPBS containing 0.1 μg/mL 4',6-diamidino-2-phenylindole (DAPI, BD Biosciences). CD8^+^ T cells that bound to pMHCI tetramer were identified using SH800 (Sony Biotechnology). PBMCs were treated with 450 nM dasatinib (Cayman Chemical) for inhibiting TCR downregulation from surface of T cells before tetramer staining [23].

## Results

### Prediction and selection of MHC class I-binding peptides derived from SARS-CoV-2 viral proteins

**Fig. 1:**
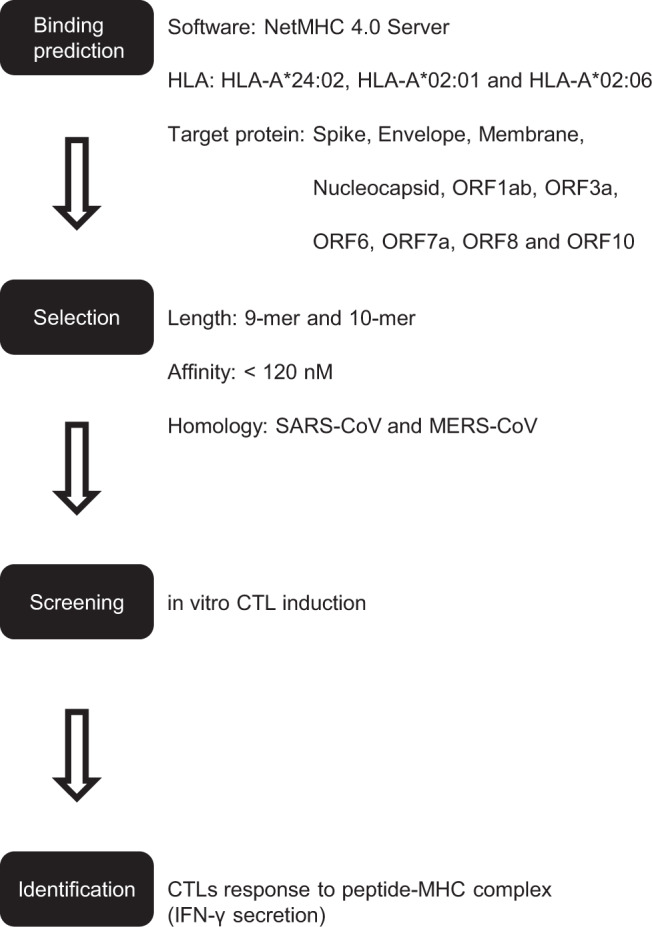
Flowchart for identifying MHC class I-binding peptides-candidates derived from SARS-CoV-2 viral proteins

The workflow for screening of SARS-CoV-2-derived MHC class I-binding peptides is shown in Fig. 1. Firstly, we predicted MHC class I-binding peptide epitopes derived from SARS-CoV-2 viral proteins including the structural proteins (spike, envelope, membrane glycoprotein, and nucleocapsid phosphoprotein) as well as the non-structural proteins (ORF1ab, ORF3a, ORF6, ORF7a, ORF8, and ORF10) as previously described [24]. Peptide-MHC affinity was calculated using NetMHC 4.0 server [25, 26]. We targeted HLA-A*24:02, HLA-A*02:01, and HLA-A*02:06 because they covered a large proportion (more than 80%) of Japanese populations [27].

We focused peptide epitopes that were conserved in either or both of SARS-CoV and Middle East respiratory syndrome coronavirus (MERS-CoV) to develop common and universal vaccines that can be possibly effective for newly arising coronaviruses in the future.

We firstly selected 14 peptides (P01-P14) that were expected to bind to an HLA-A*24:02 molecule (predicted MHC affinity of <120 nM). Two of the 14 peptides (P06 and P11) were common among coronaviruses including SARS-CoV and MERS-CoV. The remaining 12 peptides were conserved in SARS-CoV. We also selected 15 peptides (P15-P28 and P08) that were expected to bind to both HLA-A*02:01 and HLA-A*02:06 molecules (predicted MHC affinity of <120 nM). They are commonly shared with SARS-CoV. All candidate peptides are shown in Table 1.

**Table 1:** SARS-CoV-2-derived candidate peptides bind MHC class I (HLA-A*24:02, HLA-A*02:01, or HLA-A*02:06)

ID	Protein	Sequence	Length (amino acids)	Starting position	Affinity (nM)	HLA	Conserved in
P01	ORF1ab	TYASALWEI	9	4090	18	A*24:02	SARS-CoV
P02	S	PFAMQMAYRF	10	897	39	A*24:02	SARS-CoV
P03	ORF1ab	YDYLVSTQEF	10	3811	41	A*24:02	SARS-CoV
P04	ORF1ab	YYSQLMCQPI	10	2560	44	A*24:02	SARS-CoV
P05	ORF1ab	SYYSLLMPI	9	4628	46	A*24:02	SARS-CoV
P06	ORF1ab	AYANSVFNI	9	5080	56	A*24:02	SARS-CoV MERS-CoV
P07	ORF1ab	RYKLEGYAF	9	6676	62	A*24:02	SARS-CoV
P08	ORF1ab	FTYASALWEI	10	4089	69 21 5	A*24:02 A*02:01 A*02:06	SARS-CoV
P09	ORF1ab	SYSLFDMSKF	10	7039	85	A*24:02	SARS-CoV
P10	ORF1ab	RYFKYWDQTY	10	4677	90	A*24:02	SARS-CoV
P11	ORF1ab	TAYANSVFNI	10	5079	102	A*24:02	SARS-CoV MERS-CoV
P12	M	LWLLWPVTL	9	54	108	A*24:02	SARS-CoV
P13	ORF1ab	TYASALWEIQ	10	4090	111	A*24:02	SARS-CoV
P14	ORF1ab	YAYLRKHFSM	10	5138	115	A*24:02	SARS-CoV
P15	ORF1ab	SLFDMSKFPL	10	7041	6 18	A*02:01 A*02:06	SARS-CoV
P16	ORF1ab	LMYKGLPWNV	10	6077	7 8	A*02:01 A*02:06	SARS-CoV
P17	ORF1ab	FVLWAHGFEL	10	6108	8 13	A*02:01 A*02:06	SARS-CoV
P18	ORF1ab	YMPASWVMRI	10	3654	12 103	A*02:01 A*02:06	SARS-CoV
P19	ORF1ab	MMGFKMNYQV	10	5982	18 77	A*02:01 A*02:06	SARS-CoV
P20	ORF1ab	FELTSMKYFV	10	6115	18 59	A*02:01 A*02:06	SARS-CoV
P21	ORF1ab	MMILSDDAV	9	5147	35 13	A*02:01 A*02:06	SARS-CoV
P22	N	FGMSRIGMEV	10	315	37 105	A*02:01 A*02:06	SARS-CoV
P23	ORF1ab	TLMIERFVSL	10	5245	42 48	A*02:01 A*02:06	SARS-CoV
P24	ORF1ab	YVYNPFMIDV	10	6160	51 15	A*02:01 A*02:06	SARS-CoV
P25	ORF1ab	NVLAWLYAAV	10	3466	72 11	A*02:01 A*02:06	SARS-CoV
P26	ORF1ab	TLEPEYFNSV	10	5740	79 22	A*02:01 A*02:06	SARS-CoV
P27	ORF1ab	YSFLPGVYSV	10	3098	81 23	A*02:01 A*02:06	SARS-CoV
P28	ORF1ab	FMIDVQQWGF	10	6165	84 29	A*02:01 A*02:06	SARS-CoV

*S* spike protein, *M* membrane glycoprotein, *N* nucleocapsid phosphoprotein

### Identification of MHC class I-binding peptides derived from SARS-CoV-2 (HLA-A*24:02)

**Fig. 2:**
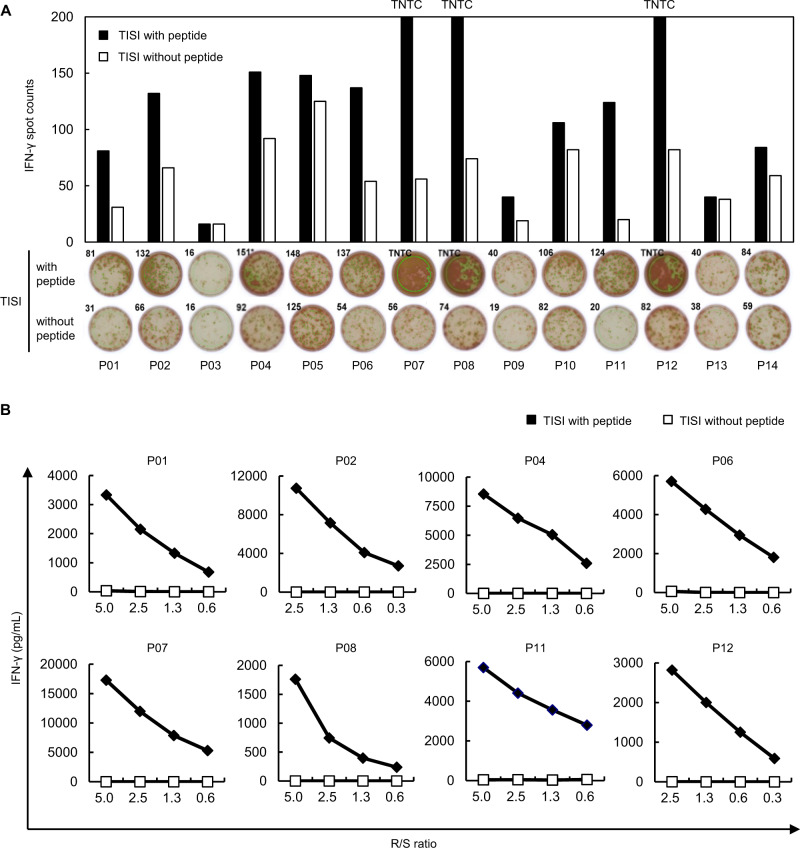
Identification of HLA-A*24:02-binding peptides derived from SARS-CoV-2. **A** IFN-γ ELISPOT assay was performed after in vitro CTL induction. CD8^+^ T cells and HLA-A*24:02-positive TISI cells pulsed with each peptide were co-cultured overnight. TISI cells without the peptide were used as negative control. Bars represent the number of IFN-γ spots. CD8^+^ T cell responses were judged as positive when the number of IFN-γ spots were 1.5 times or higher than the negative control (P01, P02, P04, P06, P07, P08, P09, P11, and P12). Representative positive activities to P02, P06, P08, and P12 observed in PBMCs from two donors are shown. No positive response was observed for P03, P05, P10, P13, and P14 in two PBMCs. Spot counts higher than 200 are demonstrated as “too numerous to count (TNTC)”. **B** To confirm the response of CTLs to SARS-CoV-2-derived peptides, IFN-γ ELISA was performed. CTLs (Responders) were co-cultured overnight with TISI cells (Stimulators) pulsed with or without the peptide at the indicated ratio of Responders to Stimulators (R/S ratio). IFN-γ secretion was measured by ELISA. CTLs that revealed the peptide-specific IFN-γ secretion for eight peptides are shown. Similar results were obtained in independent experiments using three CTLs in the respective peptides

To examined CTL induction by peptides, we first used HLA-A*24:02-positive PBMCs isolated from two healthy donors. Briefly, autologous CD8^+^ T cells and monocyte-derived DCs were prepared from PBMCs. CD8^+^ T cells were cultured with DCs pulsed with each peptide. After 11 days of stimulation by each peptide, CD8^+^ T cells were cultured overnight with HLA-A*24:02-positive TISI cells that were pulsed with or without each peptide. CD8^+^ T cell responses to each peptide were analyzed by an IFN-γ ELISPOT assay. They were considered as positive when IFN-γ spot counts were 1.5 times or higher, compared with the peptide-negative control. Positive reactivities were detected in 9 of the 14 peptides examined (Fig. 2A).

We subsequently conducted limiting dilution to establish peptide-specific CTL clones from the initial CD8^+^ T cell populations that showed positive reactivity in the IFN-γ ELISPOT assay. CD8^+^ T cells were diluted to 10 cells per well in 96-well round-bottom plates. After 14 days of the culture, peptide-specific CTLs were screened using an IFN-γ ELISPOT assay. CTL responses to each peptide were examined by an IFN-γ ELISA following the cell expansion. Peptide-specific IFN-γ secretion against eight peptides were observed when TISI cells were used as stimulator cells for presenting individual peptides (Fig. 2B). This indicates that these peptides were likely to be presented on the HLA-A*24:02 molecule and recognized by TCRs on CTLs.

### Identification of MHC class I-binding peptides derived from SARS-CoV-2 (HLA-A*02:01 and HLA-A*02:06)

**Fig. 3:**
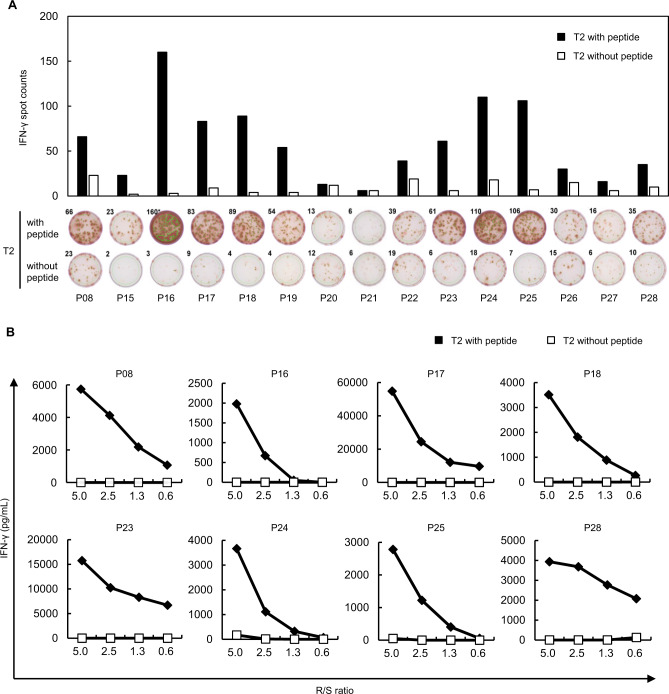
Identification of HLA-A*02:01-binding peptides derived from SARS-CoV-2. **A** IFN-γ ELISPOT assay was performed after in vitro CTL induction. CD8^+^ T cells and HLA-A*02:01-positive T2 cells pulsed with each peptide were co-cultured overnight. T2 cells without the peptide were used as negative control. Bars represent the number of IFN-γ spots. CD8^+^ T cell responses were judged as positive when the number of IFN-γ spots were 1.5 times or higher than the negative control. **B** To confirm the response of CTLs to SARS-CoV-2-derived peptides, IFN-γ ELISA was performed. CTLs (Responders) were co-cultured overnight with T2 cells (Stimulators) pulsed with or without the peptide at the indicated ratio of Responders to Stimulators (R/S ratio). IFN-γ secretion was measured by ELISA. CTLs that revealed the peptide-specific IFN-γ secretion for eight peptides are shown. Similar results were obtained in independent experiments using three CTLs in the respective peptides

We similarly investigated CTL induction for 15 candidate epitopes (P15-P28 and P08 listed in Table 1) using HLA-A*02:01-positive donor-derived PBMCs. CD8^+^ T cells were cultured overnight with HLA-A*02:01-positive T2 cells pulsed with or without each peptide. Positive reactivity was detected by an IFN-γ ELISPOT assay for 13 of the 15 peptides examined (Fig. 3A). Then, we performed limiting dilution and further confirmed the peptide-specific IFN-γ secretion in expanded T cells stimulated with 8 of the 13 peptides (Fig. 3B). Since HLA-A*02:01 and HLA-A*02:06 have an only one amino acid substitution (F9Y) in a region of the peptide binding groove [28, 29], it has been indicated that some peptides bind to both HLA-A*02:01 and HLA-A*02:06 molecules [30, 31]. Hence, we investigated the cross-reactivity of these eight peptides to an HLA-A*02:06 molecule using HLA-A*02:06-positive donor-derived PBMCs. As we expected, we found the positive reactivity of seven peptides in an IFN-γ ELISPOT assay (Fig. S1A) as well as the peptide-specific IFN-γ secretions in an ELISA (Fig. S1B).

### TCR sequences recognizing SARS-CoV-2-derived peptides

SARS-CoV-2-derived peptide-specific CTL clones were established from CD8^+^ T cell population that showed positive reactivity to each of the peptides after in vitro CTL induction (Fig. S2A, B). Then DNA sequences of pairs of TCRα and TCRβ were examined using established CTL clones. We show pairs of TRA and TRB sequences that were likely to recognize SARS-CoV-2-derived peptides in Table 2.

**Table 2:** SARS-CoV-2-derived peptide-reactive TCRs

Peptide ID		Peptide-reactive TCRs				HLA
		Chain	V region	J region	CDR3	
P04		Alpha	TRAV10	TRAJ31	CVVSALNARLMF	A*24:02
		Beta	TRBV20-1	TRBJ2-1	CSARDYTSSYNEQFF	
P06		Alpha	TRAV1-2	TRAJ32	CAVRGSGGGATNKLIF	A*24:02
		Beta	TRBV9	TRBJ2–7	CASSPSGPNYEQYF	
P07		Alpha	TRAV20	TRAJ11	CAVHFFPGYSTLTF	A*24:02
		Beta	TRBV30	TRBJ2–7	CAGAGRGYEQYF	
P08		Alpha	TRAV26-1	TRAJ5	CIVSDMGRRALTF	A*24:02
		Beta	TRBV4-1	TRBJ2-1	CASSFTGTSGSLGEQFF	
P11		Alpha	TRAV21	TRAJ20	CAVFNDYKLSF	A*24:02
		Beta	TRBV7–9	TRBJ2–7	CASSSRTSGRSSYEQYF	
P08		Alpha	TRAV21	TRAJ48	CAVRRTYFGNEKLTF	A*02:01
		Beta	TRBV20-1	TRBJ2–7	CSARGSDSYEQYF	
P23		Alpha	TRAV8-6	TRAJ36	CAITGANNLFF	A*02:01
		Beta	TRBV20-1	TRBJ1–5	CSAEDRGSNSNQPQHF	
P25	**1**	Alpha	TRAV1-2	TRAJ20	CAVRDPRNDYKLSF	A*02:01
		Beta	TRBV27	TRBJ1-1	CATQVNTEAFF	
	**2**	Alpha	TRAV24	TRAJ29	CAFLSGNTPLVF	A*02:01
		Beta	TRBV27	TRBJ1–6	CASSPAAYSPLHF	

### Detection of SARS-CoV-2-derived peptides-reactive CD8^+^ T cells in PBMCs

**Fig. 4:**
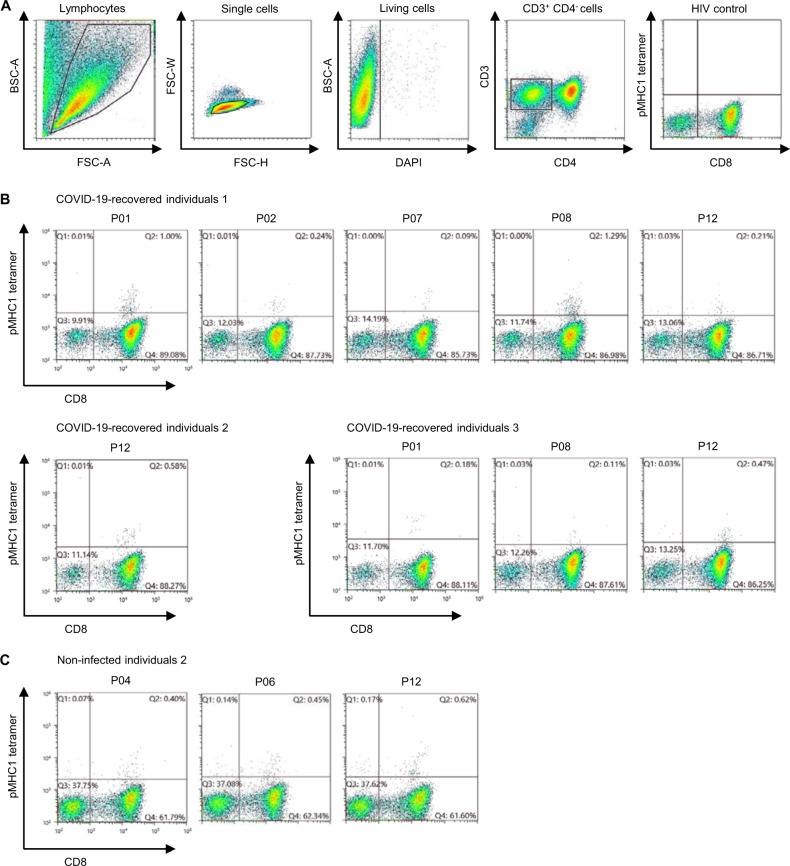
Detection of CD8^+^ T cells that recognized SARS-CoV-2-derived MHC class I-binding peptides in PBMCs. **A** Gating strategy for detecting SARS-CoV-2-derived peptides-reactive CD8^+^ T cells. Firstly, lymphocytes population was separated by a forward scatter (FSC-A) and back scatter (BSC-A) gates. Doublets were excluded on forward scatter height (FSC-H) and forward scatter width (FSC-W). Living CD3^+^ CD4^−^ cells were selected by staining with DAPI, and expression of CD3 and CD4. pMHC1 tetramer-binding CD8^+^ T cells were defined by gating based on HIV control tetramer staining. **B** HLA-A*24:02-positive PBMCs from three COVID-19-recovered individuals were stained using pMHCI tetramers after in vitro stimulation. CD8^+^ T cells that recognize SARS-CoV-2-derived peptides were detected from every individual. **C** HLA-A*24:02-positive PBMCs from three healthy individuals without any history of SARS-CoV-2 infection were stained using pMHCI tetramers after in vitro stimulation. Pre-existing CD8^+^ T cells that recognize SARS-CoV-2-derived peptides were detected from one of the three non-infected individuals

We analyzed HLA-A*24:02-positive PBMCs from individuals who recovered from COVID-19 (*n* = 3) and healthy individuals without any evident SARS-CoV-2 infection (*n* = 3) to examine CD8^+^ T cells that might recognize MHC class I-binding peptides we identified. After in vitro stimulation, PBMCs were stained with PE-conjugated pMHCI tetramers and analyzed by flow cytometry (Fig. 4A). pMHCI tetramer-binding CD8^+^ T cells were detected from three individuals recovered from COVID-19 (Fig. 4B). It is notable that we found CD8^+^ T cells recognizing SARS-CoV-2-derived peptides (P04, P06, and P12), which are conserved in other coronaviruses (Table S1), in one non-infected healthy individual as well (Fig. 4C).

### Impact of SARS-CoV-2 omicron variant on MHC class I-binding peptides

We investigated the influence of mutations found in SARS-CoV-2 omicron variant (BA.1), which is a new SARS-CoV-2 variant of concern and has been identified in 89 countries as of 16th of December, 2021, on 15 MHC class I-binding peptides identified in this study. We searched nonsynonymous substitutions and deletions in the GISAID database (https://www.gisaid.org) on 20th of December, 2021. We identified 132 mutations (76 mutations in ORF1ab polyprotein, 51 mutations in spike protein, and 5 mutations in membrane glycoprotein) in 9 or more (>0.1%) of 8993 sequences of omicron variant. Only one mutation at position 5086 (F5086Y) in ORF1ab may affect immunogenicity of P06 and P11, but other peptide sequences are conserved in the omicron variant (Table S2). This indicates that the peptides identified in this study may be used as a sort of universal peptides to induce CTLs against COVID-19 variants.

## Discussion

We screened 29 SARS-CoV-2-derived epitope peptides (14 peptides for HLA-A*24:02 and 15 peptides for HLA-A*02:01) that were predicted to bind to MHC class I molecules for activation of CD8^+^ T cells and found that eight peptides each for HLA-A*24:02 and HLA-A*02:01 could activate CD8^+^ T cells isolated from PBMCs. We subsequently established CTL clones and confirmed their immune reactivities against these peptides presented on the expected HLA molecules. Among the eight peptides that activated CD8^+^ T cells derived from HLA-A*02:01 donors, seven peptides also activated CD8^+^ T cells derived from HLA-A*02:06-positive PBMCs. Interestingly, one peptide (P08) commonly induced CTLs derived from all of HLA-A*24:02-, HLA-A*02:01-, and HLA-A*02:06-positive PBMCs. Hence, a total of 15 peptides were likely to be presented on MHC class I molecules and activated CD8^+^ T cells. These 15 peptides corresponded to parts of spike protein, membrane glycoprotein, or ORF1ab polyprotein (Fig. S3).

Variant SARS-CoV-2 strains, alpha, beta, gamma, delta, and omicron, which are now classified as variants of concern by World Health Organization, rapidly expanded worldwide and are still expanding in some countries. SARS-CoV-2 variants of concern appear to escape at some extent from vaccinations using mRNA encoding the spike protein because they have mutations in domains recognized by neutralizing antibodies. Since multiple peptides identified from spike protein, membrane glycoprotein, and ORF1ab polyprotein in this study are well conserved among coronavirus species, it is likely that these regions are essential for virus survival or expansion. Hence, T cells induced by these peptides reported here might be less affected by mutations that may additionally occur in various virus proteins. In fact, mutations found in the omicron variant have little effect on immunogenicity of these peptides at present although we need to further monitor the variant sequences.

We confirmed the presence of CD8^+^ T cells that recognize SARS-CoV-2-derived peptides (P01, P02, P07, P08, and P12) in HLA-A*24:02-positive individuals recovered from COVID-19, indicating that CD8^+^ T cells were activated in vivo by coronavirus-derived peptides (possibly same as peptides identified in this study) presented on MHC class I molecules on antigen presenting cells including DCs in their body. It is notable that we found CD8^+^ T cells that recognized SARS-CoV-2-derived peptides (P04, P06, and P12) in one SARS-CoV-2-non-infected healthy individual. Since these peptides are conserved among other human coronaviruses, the presence of CD8^+^ T cells reacting to these peptides may reflect previous infections with these viruses, or this individual might have experienced non-symptomatic COVID-19. Understanding the effect of pre-existing SARS-CoV-2-derived peptide-reactive CD8^+^ T cells on non-symptomatic patients or prevention of COVID-19 should be important to consider the strategy for controlling pandemic or the future threat to newly arising coronaviruses.

Recently, one report indicated that a spike protein-derived polypeptide with adjuvant on nanoparticle, which was administrated by intramuscular route, successfully induced neutralizing antibodies and CD4^+^ T cell responses in rhesus macaques [32]. This report also supports the concept that SARS-CoV-2-derived epitope peptides might be a promising COVID-19 vaccine that would induce and maintain the T cell immunity against SARS-CoV-2 that will continuously cause new mutations. In addition, we found no significant homology with peptides possibly derived from human proteins by the BLAST algorithm (https://blast.ncbi.nlm.nih.gov/Blast.cgi), suggesting there is little possibility that our peptides might cause unintended immune reactions, which are unfavorable to our health condition.

Furthermore, T-Detect COVID Test (Adaptive Biotechnologies), a next-generation TCR sequencing-based test, was approved by FDA for identifying the recent or prior SARS-CoV-2 infection. Our peptide-reactive TCR sequences might be useful to investigate SARS-CoV-2 infection history or monitor the effectiveness in vaccinated individuals.

In this study, we have reported identification of 15 candidate vaccine peptides against COVID-19. Since T cell memories seem to be maintained in a longer period (few to several years) than antibodies and the peptides reported here are likely to avoid the influence of additional mutations that may occur in SARS-CoV-2 in the future, our peptides may enable us to provide the useful information to design the novel vaccine(s).

## Supplementary information

Supplementary Information

Conflict of Interest
