# Supplementary material for: Identification of cytotoxic T cells and their T cell receptor sequences targeting COVID-19 using MHC class I-binding peptides
Source: J Hum Genet. 2022 Feb 2;67(7):411–9. doi: 10.1038/s10038-022-01013-4 (PMC8807680; doi:10.1038/s10038-022-01013-4)
Supplement: Supplementary file 1 — Supplementary Information [file 10038_2022_1013_MOESM1_ESM.docx]

Supplementary Information for

Identification of cytotoxic T cells and their T cell receptor sequences targeting COVID-19 using MHC class I-binding peptides

Tetsuro Hikichi,* Michiko Sakamoto, Makiko Harada, Maki Saito, Yuka Yamane, Kimihisa Tokumura, Yusuke Nakamura

*Corresponding author. Email: t-hikichi@oncotherapy.co.jp

**This file includes:**

Fig. S1. Identification of HLA-A*02:06-binding peptides derived from SARS-CoV-2

Fig. S2. Establishment of peptide-specific CTL clones

Fig. S3. Locations of epitope peptides identified in this study, corresponding to SARS-CoV-2 viral proteins

Table S1. List of SARS-CoV-2-derived peptide sequences and their homology with other human coronaviruses

Table S2. List of mutations of SARS-CoV-2 omicron variant.


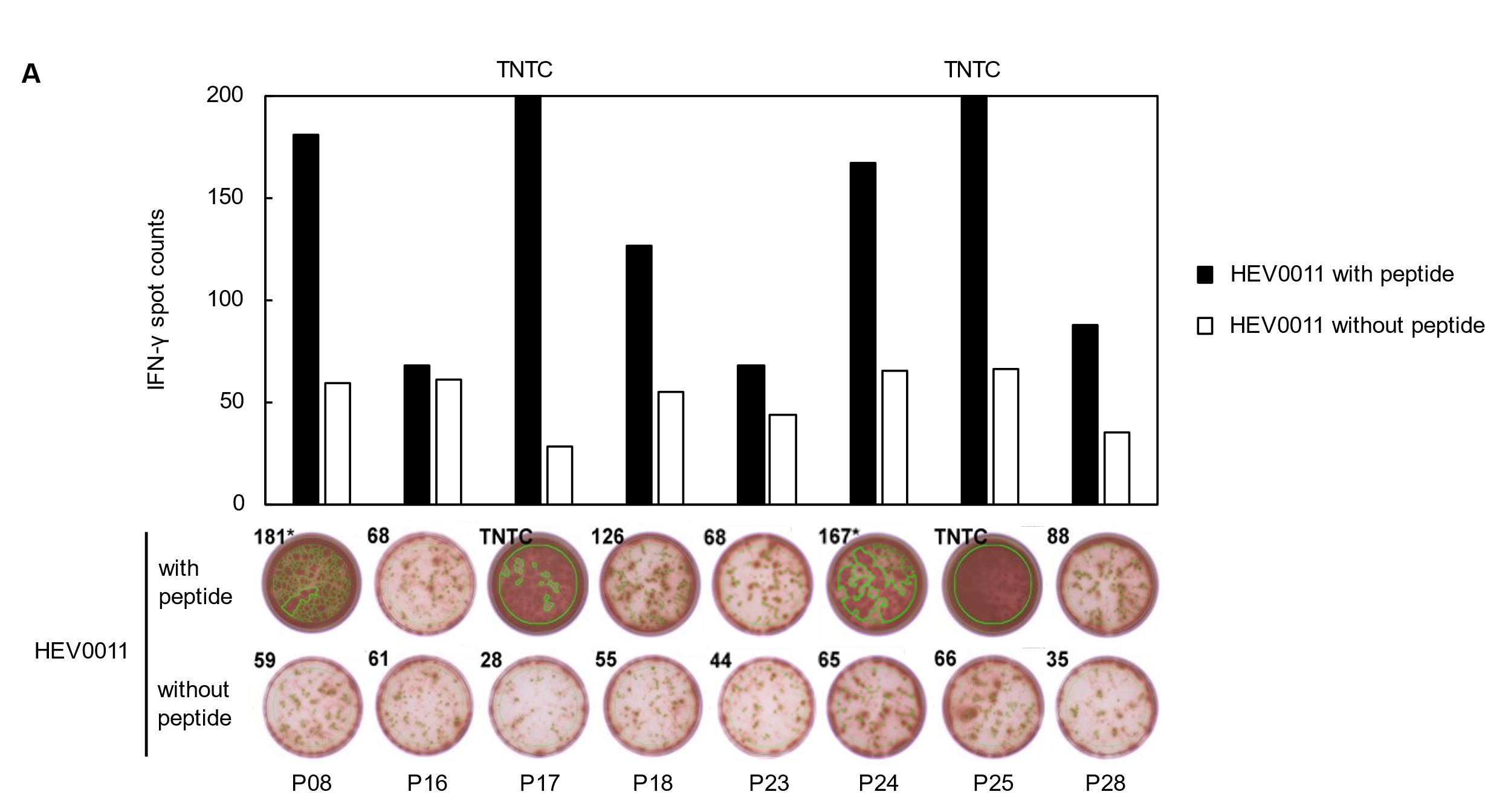


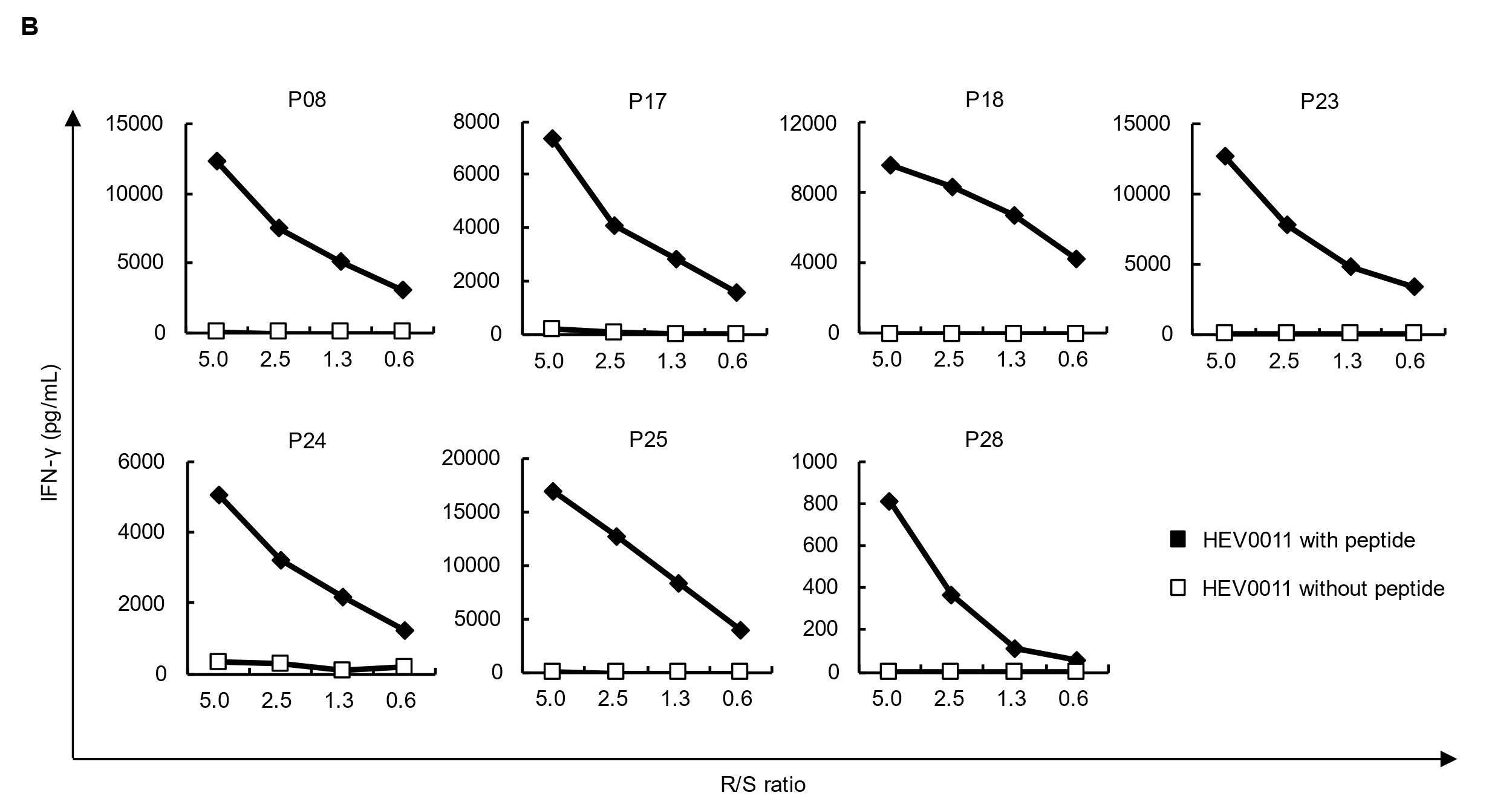


**Fig. S1.** **Identification of HLA-A*02:06-binding peptides derived from SARS-CoV-2.** (**A**) IFN-γ ELISPOT assay was performed after *in vitro* CTL induction. CD8^+^ T cells and HLA-A*02:06-positive HEV0011 cells pulsed with each peptide were co-cultured overnight. HEV0011 cells without the peptide were used as negative control. Bars represent the number of IFN-γ spots. CD8^+^ T cell responses were judged as positive when the number of IFN-γ spots were 1.5 times or higher than the negative control. In this study, spot counts higher than 200 are demonstrated as “too numerous to count (TNTC)”. (**B**) To confirm the response of CTLs to SARS-CoV-2-derived peptides, IFN-γ ELISA was performed. CTLs (Responders) were co-cultured overnight with HEV0011 cells (Stimulators) pulsed with or without the peptide at the indicated ratio of Responders to Stimulators (R/S ratio). IFN-γ secretion was measured by ELISA. CTLs that revealed the peptide-specific IFN-γ secretion for seven peptides are shown. Similar results were obtained in independent experiments using three CTLs in the respective peptides.


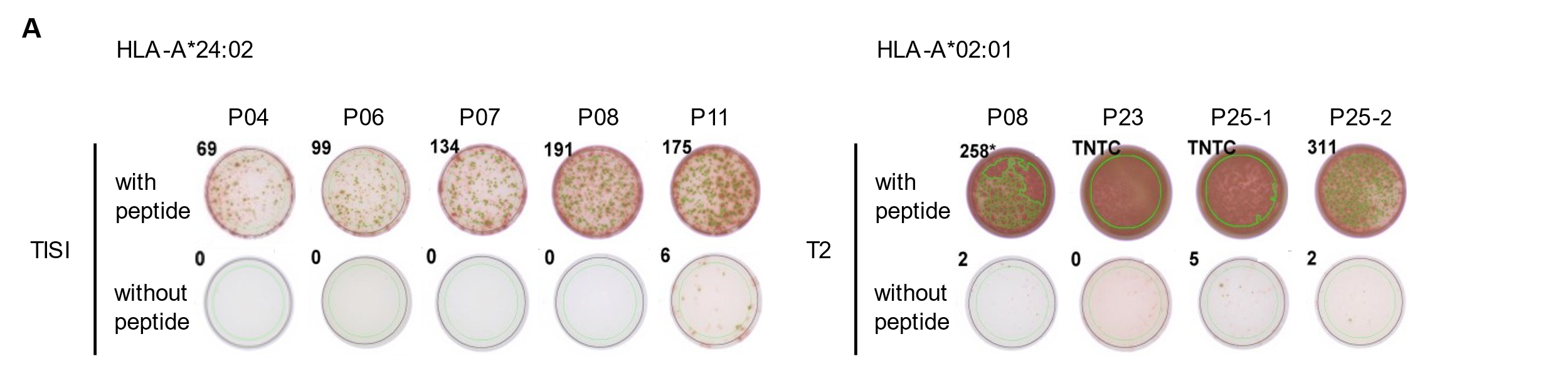


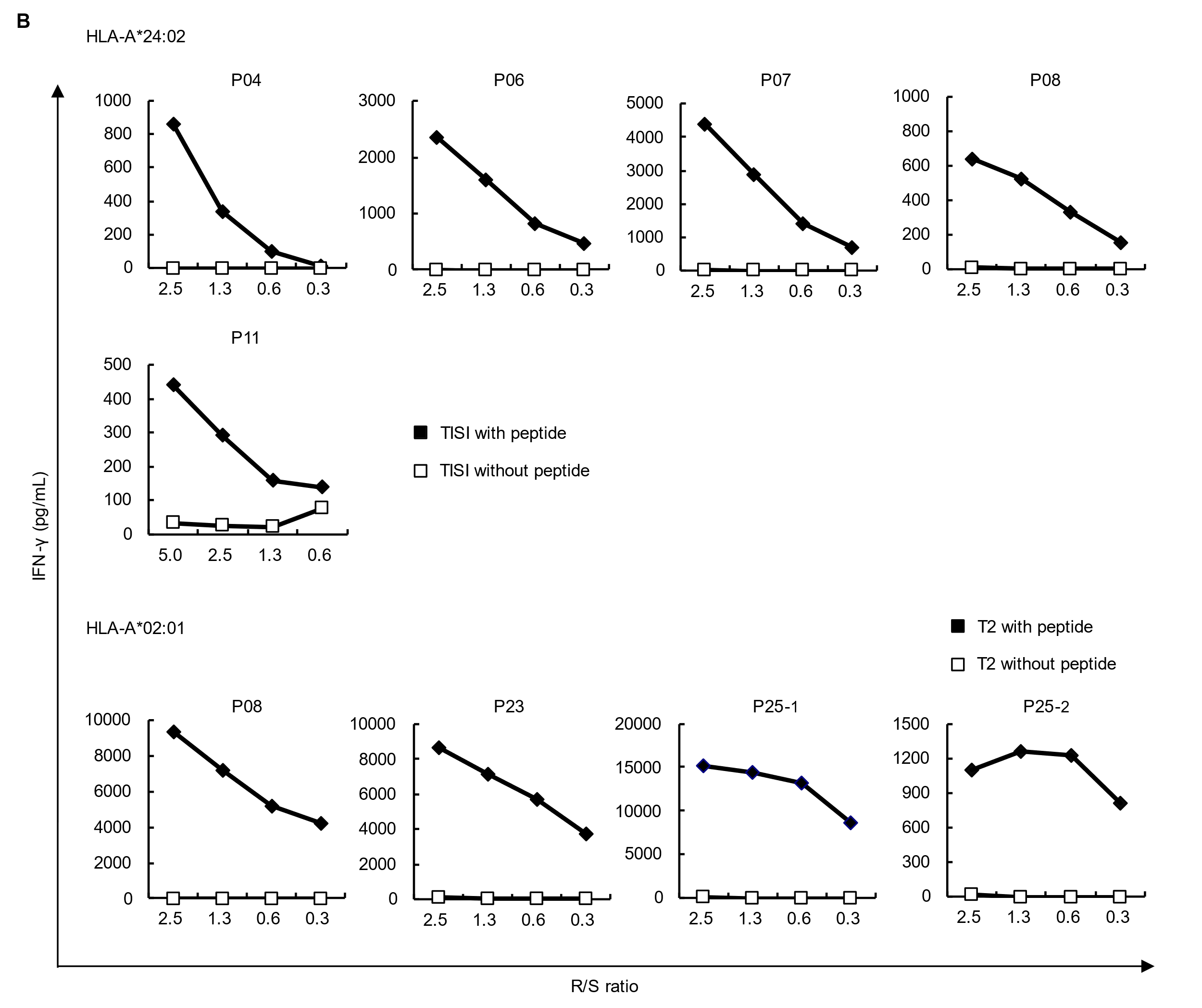


**Fig. S2. Establishment of peptide-specific CTL clones.** SARS-CoV-2-derived peptide-specific CTL clones were established from HLA-A*24:02- or HLA-A*02:01-positive donor-derived PBMCs. After *in vitro* CTL induction, CD8^+^ T cells were diluted to 0.5 cell per well in 96-well round-bottom plates (limiting dilution) and further cultured. (**A**) Peptide-specific CTL clones were screened by an IFN-γ ELISPOT assay after limiting dilution. (**B**) CTL clones revealing SARS-CoV-2-derived peptide-specific IFN-γ secretion in an ELISA after expansion culture are shown. We established two CTL clones that recognize P25.


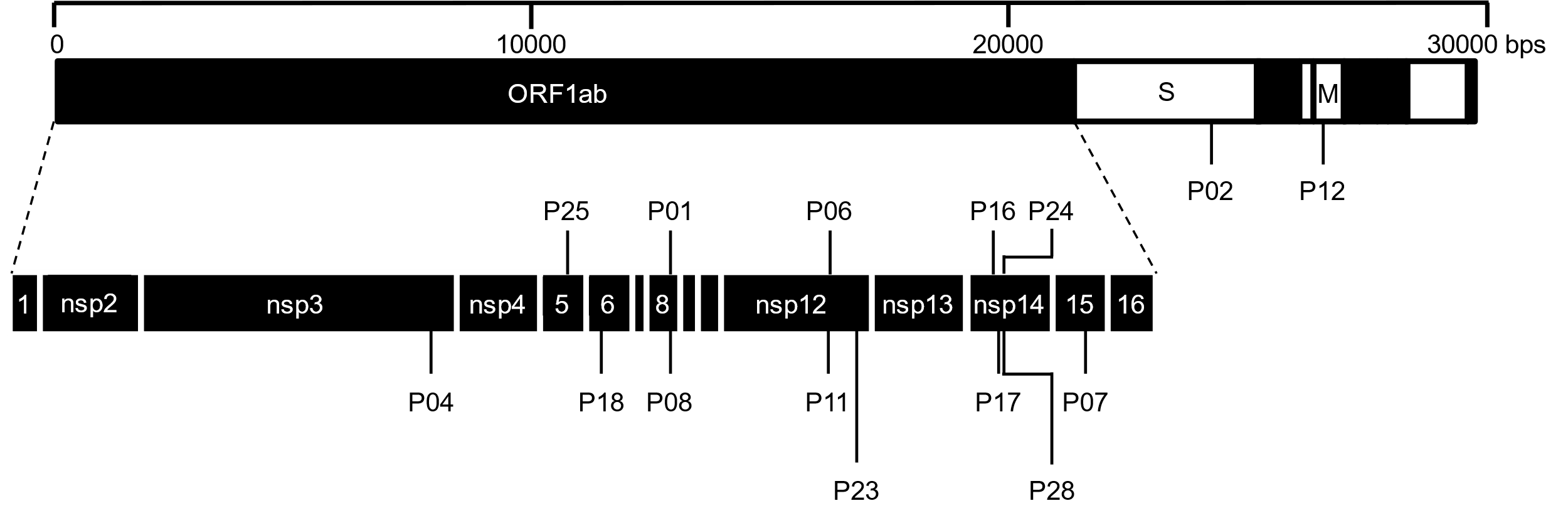


**Fig. S3. Locations of epitope peptides identified in this study, corresponding to SARS-CoV-2 viral proteins.** Abbreviations: S, spike protein; M, membrane glycoprotein; nsp, nonstructural protein.

**Table S1. List of SARS-CoV-2-derived peptide sequences and their homology with other human coronaviruses.**

| Coronavirus | Amino acid sequence in peptide | | |
| --- | --- | --- | --- |
| SARS-CoV-2 | YYSQLMCQPI (P04) | AYANSVFNI (P06) | LWLLWPVTL (P12) |
| SARS-CoV | YYSQLMCQPI (P04) | AYANSVFNI (P06) | LWLLWPVTL (P12) |
| MERS-CoV | - | AYANSVFNI (P06) | - |
| HCoV-229E | - | AYANSVFNI (P06) | - |
| HCoV-NL63 | - | - | - |
| HCoV-HKU1 | - | - | - |
| HCoV-OC43 | - | - | - |

Minus (-) means no significant homology (amino acid sequence identity is less than 100%).

**Table S2. List of mutations of SARS-CoV-2 omicron variant.**

| Protein | Mutation | MHC class I-binding peptides | | | | | | | | | | | | | | |
| --- | --- | --- | --- | --- | --- | --- | --- | --- | --- | --- | --- | --- | --- | --- | --- | --- |
|  |  | **P01** | **P02** | **P04** | **P06** | **P07** | **P08** | **P11** | **P12** | **P16** | **P17** | **P18** | **P23** | **P24** | **P25** | **P28** |
| ORF1ab polyprotein | R24C | - | - | - | - | - | - | - | - | - | - | - | - | - | - | - |
|  | 82-86 del | - | - | - | - | - | - | - | - | - | - | - | - | - | - | - |
|  | G112S | - | - | - | - | - | - | - | - | - | - | - | - | - | - | - |
|  | 141-143 del | - | - | - | - | - | - | - | - | - | - | - | - | - | - | - |
|  | S142L | - | - | - | - | - | - | - | - | - | - | - | - | - | - | - |
|  | H374Y | - | - | - | - | - | - | - | - | - | - | - | - | - | - | - |
|  | K564N | - | - | - | - | - | - | - | - | - | - | - | - | - | - | - |
|  | G697R | - | - | - | - | - | - | - | - | - | - | - | - | - | - | - |
|  | A735T | - | - | - | - | - | - | - | - | - | - | - | - | - | - | - |
|  | **K856R** | - | - | - | - | - | - | - | - | - | - | - | - | - | - | - |
|  | Y947H | - | - | - | - | - | - | - | - | - | - | - | - | - | - | - |
|  | D983G | - | - | - | - | - | - | - | - | - | - | - | - | - | - | - |
|  | T999I | - | - | - | - | - | - | - | - | - | - | - | - | - | - | - |
|  | T1004I | - | - | - | - | - | - | - | - | - | - | - | - | - | - | - |
|  | L1130F | - | - | - | - | - | - | - | - | - | - | - | - | - | - | - |
|  | P1147S | - | - | - | - | - | - | - | - | - | - | - | - | - | - | - |
|  | M1193V | - | - | - | - | - | - | - | - | - | - | - | - | - | - | - |
|  | I1203T | - | - | - | - | - | - | - | - | - | - | - | - | - | - | - |
|  | A1352V | - | - | - | - | - | - | - | - | - | - | - | - | - | - | - |
|  | R1628H | - | - | - | - | - | - | - | - | - | - | - | - | - | - | - |
|  | P1786L | - | - | - | - | - | - | - | - | - | - | - | - | - | - | - |
|  | P1803S | - | - | - | - | - | - | - | - | - | - | - | - | - | - | - |
|  | T1822I | - | - | - | - | - | - | - | - | - | - | - | - | - | - | - |
|  | A1824V | - | - | - | - | - | - | - | - | - | - | - | - | - | - | - |
|  | L1853F | - | - | - | - | - | - | - | - | - | - | - | - | - | - | - |
|  | V1887I | - | - | - | - | - | - | - | - | - | - | - | - | - | - | - |
|  | **S2083I** | - | - | - | - | - | - | - | - | - | - | - | - | - | - | - |
|  | **2084 del** | - | - | - | - | - | - | - | - | - | - | - | - | - | - | - |
|  | T2152I | - | - | - | - | - | - | - | - | - | - | - | - | - | - | - |
|  | S2285F | - | - | - | - | - | - | - | - | - | - | - | - | - | - | - |
|  | S2488F | - | - | - | - | - | - | - | - | - | - | - | - | - | - | - |
|  | **A2710T** | - | - | - | - | - | - | - | - | - | - | - | - | - | - | - |
|  | M2796T | - | - | - | - | - | - | - | - | - | - | - | - | - | - | - |
|  | V2857A | - | - | - | - | - | - | - | - | - | - | - | - | - | - | - |
|  | **T3255I** | - | - | - | - | - | - | - | - | - | - | - | - | - | - | - |
|  | V3261I | - | - | - | - | - | - | - | - | - | - | - | - | - | - | - |
|  | **P3395H** | - | - | - | - | - | - | - | - | - | - | - | - | - | - | - |
|  | P3395Y | - | - | - | - | - | - | - | - | - | - | - | - | - | - | - |
|  | L3606F | - | - | - | - | - | - | - | - | - | - | - | - | - | - | - |
|  | **3674-3676 del** | - | - | - | - | - | - | - | - | - | - | - | - | - | - | - |
|  | 3674-3675 del | - | - | - | - | - | - | - | - | - | - | - | - | - | - | - |
|  | 3675-3676 del | - | - | - | - | - | - | - | - | - | - | - | - | - | - | - |
|  | **I3758V** | - | - | - | - | - | - | - | - | - | - | - | - | - | - | - |
|  | V3917I | - | - | - | - | - | - | - | - | - | - | - | - | - | - | - |
|  | Q4015R | - | - | - | - | - | - | - | - | - | - | - | - | - | - | - |
|  | T4158I | - | - | - | - | - | - | - | - | - | - | - | - | - | - | - |
|  | T4174I | - | - | - | - | - | - | - | - | - | - | - | - | - | - | - |
|  | G4178C | - | - | - | - | - | - | - | - | - | - | - | - | - | - | - |
|  | D4200A | - | - | - | - | - | - | - | - | - | - | - | - | - | - | - |
|  | T4249I | - | - | - | - | - | - | - | - | - | - | - | - | - | - | - |
|  | G4436S | - | - | - | - | - | - | - | - | - | - | - | - | - | - | - |
|  | D4545Y | - | - | - | - | - | - | - | - | - | - | - | - | - | - | - |
|  | I4615V | - | - | - | - | - | - | - | - | - | - | - | - | - | - | - |
|  | T4618M | - | - | - | - | - | - | - | - | - | - | - | - | - | - | - |
|  | **P4715L** | - | - | - | - | - | - | - | - | - | - | - | - | - | - | - |
|  | F5086Y | - | - | - | ● | - | - | ● | - | - | - | - | - | - | - | - |
|  | Q5267R | - | - | - | - | - | - | - | - | - | - | - | - | - | - | - |
|  | Y5593C | - | - | - | - | - | - | - | - | - | - | - | - | - | - | - |
|  | T5805M | - | - | - | - | - | - | - | - | - | - | - | - | - | - | - |
|  | A5877T | - | - | - | - | - | - | - | - | - | - | - | - | - | - | - |
|  | **I5967V** | - | - | - | - | - | - | - | - | - | - | - | - | - | - | - |
|  | P5968S | - | - | - | - | - | - | - | - | - | - | - | - | - | - | - |
|  | D6315E | - | - | - | - | - | - | - | - | - | - | - | - | - | - | - |
|  | R6316G | - | - | - | - | - | - | - | - | - | - | - | - | - | - | - |
|  | Y6317C | - | - | - | - | - | - | - | - | - | - | - | - | - | - | - |
|  | P6318H | - | - | - | - | - | - | - | - | - | - | - | - | - | - | - |
|  | Q6648H | - | - | - | - | - | - | - | - | - | - | - | - | - | - | - |
|  | A6669V | - | - | - | - | - | - | - | - | - | - | - | - | - | - | - |
|  | T6833I | - | - | - | - | - | - | - | - | - | - | - | - | - | - | - |
|  | L6924F | - | - | - | - | - | - | - | - | - | - | - | - | - | - | - |
|  | K6958R | - | - | - | - | - | - | - | - | - | - | - | - | - | - | - |
|  | P7013L | - | - | - | - | - | - | - | - | - | - | - | - | - | - | - |
|  | R7014N | - | - | - | - | - | - | - | - | - | - | - | - | - | - | - |
|  | S7089C | - | - | - | - | - | - | - | - | - | - | - | - | - | - | - |
|  | S7090R | - | - | - | - | - | - | - | - | - | - | - | - | - | - | - |
|  | D7091S | - | - | - | - | - | - | - | - | - | - | - | - | - | - | - |
| Spike protein | L5F | - | - | - | - | - | - | - | - | - | - | - | - | - | - | - |
|  | L18P | - | - | - | - | - | - | - | - | - | - | - | - | - | - | - |
|  | **A67V** | - | - | - | - | - | - | - | - | - | - | - | - | - | - | - |
|  | **69-70 del** | - | - | - | - | - | - | - | - | - | - | - | - | - | - | - |
|  | 86 del | - | - | - | - | - | - | - | - | - | - | - | - | - | - | - |
|  | **T95I** | - | - | - | - | - | - | - | - | - | - | - | - | - | - | - |
|  | L141F | - | - | - | - | - | - | - | - | - | - | - | - | - | - | - |
|  | **G142D** | - | - | - | - | - | - | - | - | - | - | - | - | - | - | - |
|  | 142-144 del | - | - | - | - | - | - | - | - | - | - | - | - | - | - | - |
|  | **143-145 del** | - | - | - | - | - | - | - | - | - | - | - | - | - | - | - |
|  | V193L | - | - | - | - | - | - | - | - | - | - | - | - | - | - | - |
|  | **N211I** | - | - | - | - | - | - | - | - | - | - | - | - | - | - | - |
|  | 211 del | - | - | - | - | - | - | - | - | - | - | - | - | - | - | - |
|  | **212 del** | - | - | - | - | - | - | - | - | - | - | - | - | - | - | - |
|  | Q218H | - | - | - | - | - | - | - | - | - | - | - | - | - | - | - |
|  | E309Q | - | - | - | - | - | - | - | - | - | - | - | - | - | - | - |
|  | **G339D** | - | - | - | - | - | - | - | - | - | - | - | - | - | - | - |
|  | R346K | - | - | - | - | - | - | - | - | - | - | - | - | - | - | - |
|  | **S371L** | - | - | - | - | - | - | - | - | - | - | - | - | - | - | - |
|  | **S373P** | - | - | - | - | - | - | - | - | - | - | - | - | - | - | - |
|  | **S375F** | - | - | - | - | - | - | - | - | - | - | - | - | - | - | - |
|  | K417N | - | - | - | - | - | - | - | - | - | - | - | - | - | - | - |
|  | N440K | - | - | - | - | - | - | - | - | - | - | - | - | - | - | - |
|  | G446S | - | - | - | - | - | - | - | - | - | - | - | - | - | - | - |
|  | L452R | - | - | - | - | - | - | - | - | - | - | - | - | - | - | - |
|  | **S477N** | - | - | - | - | - | - | - | - | - | - | - | - | - | - | - |
|  | **T478K** | - | - | - | - | - | - | - | - | - | - | - | - | - | - | - |
|  | **E484A** | - | - | - | - | - | - | - | - | - | - | - | - | - | - | - |
|  | E484V | - | - | - | - | - | - | - | - | - | - | - | - | - | - | - |
|  | **Q493R** | - | - | - | - | - | - | - | - | - | - | - | - | - | - | - |
|  | **G496S** | - | - | - | - | - | - | - | - | - | - | - | - | - | - | - |
|  | **Q498R** | - | - | - | - | - | - | - | - | - | - | - | - | - | - | - |
|  | **N501Y** | - | - | - | - | - | - | - | - | - | - | - | - | - | - | - |
|  | **Y505H** | - | - | - | - | - | - | - | - | - | - | - | - | - | - | - |
|  | **T547K** | - | - | - | - | - | - | - | - | - | - | - | - | - | - | - |
|  | **D614G** | - | - | - | - | - | - | - | - | - | - | - | - | - | - | - |
|  | **H655Y** | - | - | - | - | - | - | - | - | - | - | - | - | - | - | - |
|  | **N679K** | - | - | - | - | - | - | - | - | - | - | - | - | - | - | - |
|  | **P681H** | - | - | - | - | - | - | - | - | - | - | - | - | - | - | - |
|  | P681R | - | - | - | - | - | - | - | - | - | - | - | - | - | - | - |
|  | A701V | - | - | - | - | - | - | - | - | - | - | - | - | - | - | - |
|  | **N764K** | - | - | - | - | - | - | - | - | - | - | - | - | - | - | - |
|  | **D796Y** | - | - | - | - | - | - | - | - | - | - | - | - | - | - | - |
|  | **N856K** | - | - | - | - | - | - | - | - | - | - | - | - | - | - | - |
|  | D950N | - | - | - | - | - | - | - | - | - | - | - | - | - | - | - |
|  | **Q954H** | - | - | - | - | - | - | - | - | - | - | - | - | - | - | - |
|  | **N969K** | - | - | - | - | - | - | - | - | - | - | - | - | - | - | - |
|  | **L981F** | - | - | - | - | - | - | - | - | - | - | - | - | - | - | - |
|  | I1081V | - | - | - | - | - | - | - | - | - | - | - | - | - | - | - |
|  | D1084E | - | - | - | - | - | - | - | - | - | - | - | - | - | - | - |
|  | V1264M | - | - | - | - | - | - | - | - | - | - | - | - | - | - | - |
| Membrane glycoprotein | **D3G** | - | - | - | - | - | - | - | - | - | - | - | - | - | - | - |
|  | **Q19E** | - | - | - | - | - | - | - | - | - | - | - | - | - | - | - |
|  | L29F | - | - | - | - | - | - | - | - | - | - | - | - | - | - | - |
|  | **A63T** | - | - | - | - | - | - | - | - | - | - | - | - | - | - | - |
|  | I82T | - | - | - | - | - | - | - | - | - | - | - | - | - | - | - |

Data source is outbreak.info (<https://outbreak.info/>) based on data from GISAID database (https://www.gisaid.org/) as of 20^th^ of December, 2021. Mutations, which are defined as nonsynonymous substitutions and deletions detected in 0.1% or more of 8,993 sequences of omicron variant (BA.1), are listed. Characteristic mutations that were detected in at least 75.0% of the sequences were marked in bold. Closed circle means that peptide sequence contains the mutation site in viral proteins of omicron variant. Minus (-) represents no mutation sites in the peptide sequence.
