# Supplementary material for: Identification of cytotoxic T cells and their T cell receptor sequences targeting COVID-19 using MHC class I-binding peptides
Source: J Hum Genet. 2022 Feb 2;67(7):411–9. doi: 10.1038/s10038-022-01013-4 (PMC8807680; doi:10.1038/s10038-022-01013-4)
Supplement: Supplementary file 2 — Conflict of Interest [file 10038_2022_1013_MOESM2_ESM.pdf]

Self-reported Potential Conflict of Interest of Authors

The following relationships must be disclosed to the JHG editorial office:

1. Employment. The name and nature of all employers must be disclosed.

   □no, ■yes (name of employers: TH, MS, MH, MS, YY and KT are employees of OncoTherapy Science, Inc.)

2. Membership on the board of directors or any fiduciary relationship with another organization.

   ■no, □yes (name of organization:)

3. Membership on a scientific advisory panel or other standing scientific/medical committees of another organization.

   ■no, □yes (name of organization:)

These authors, their spouse, their relatives in the first degree, or persons who share income/assets with authors have the potential conflict of interest described below. The lower limits of money (April to March of the most recent one year) which should be reported are demonstrated in JHG's conflict of interest policy.

	Leadership Position/Advisory Role	Stock	Royalty	Lecture's Fee	Manuscript Fee	Research Funds	Other
Yen amount	≧1,000,000	≧1,000,000	≧1,000,000	≧500,000	≧500,000	≧1,000,000	≧50,000
Tetsuro Hikichi<br>Michiko Sakamoto<br>Makiko Harada<br>Maki Saito<br>Yuka Yamane<br>Kimihisa Tokumura<br>Yusuke Nakamura	OncoTherapy Science, Inc.	OncoTherapy Science, Inc.					

＊ ¥1,000,000-4,999,999　　＊＊ ¥5,000,000-9,999,999　　# ¥10,000,000≦
